# Evaluación del proceso de incapacidad temporal por contingencias comunes en el periodo prepandemia por COVID-19 (bienio 2018 y 2019)

**DOI:** 10.1016/j.aprim.2025.103282

**Published:** 2025-06-17

**Authors:** Ángel Carlos Matía Cubillo, Francisco Casanova Gómez, María Esther Cubo Delgado, Juan Valencia Ramos, Miren Elízari Roncal, Belén Angulo Fernández de Larrea

**Affiliations:** a Equipo de Atención Primaria, Centro de Salud Ignacio López Saiz, Burgos, España; b Equipo de Atención Primaria, Centro de Salud Casa la Vega, Burgos, España; c Hospital Universitario de Burgos, Burgos, España; d Equipo de Atención Primaria, Centro de Salud Burgos Rural Sur, Burgos, España

**Keywords:** **(DeCS)**, Atención primaria de salud, Médicos de atención primaria, Incapacidad laboral transitoria, Reinserción al trabajo, Condiciones de trabajo, Absentismo laboral, **(MeSH)**, Primary health care, Physicians, Primary care, Sick leave, Return to work, Working conditions, Absenteeism

## Abstract

**Objetivos:**

Conocer las características del proceso de incapacidad temporal (IT), motivos, duración real y estimada, y factores relacionados.

**Diseño:**

Estudio descriptivo, transversal y retrospectivo.

**Emplazamiento:**

Centros de salud del Área de Salud de Burgos.

**Participantes:**

Trabajadores activos, seleccionados por muestreo aleatorio simple, entre los que tuvieron un proceso de IT en 2018 y 2019.

**Mediciones principales:**

Variables sociodemográficas; relacionadas con motivos de IT, duración estimada y observada y puesto de trabajo; variables clínicas: hábitos, comorbilidades, polifarmacia y copago.

**Resultados:**

Novecientos treinta y tres trabajadores***,*** 52,4% varones, edad 43,6 ± 11,4 años. Los procesos con mayor diferencia entre duración observada y estimada fueron neoplasias, enfermedades del aparato circulatorio, trastornos mentales, sistema nervioso y musculoesqueléticas. Diferencia significativa para comorbilidades previas, polifarmacia, sin copago y > 55 años. Solicitaron pruebas complementarias el 31,8%, el 3,1% se hicieron en mutuas; e interconsultas el 33,3%. El mayor porcentaje de IT en puestos laborales no cualificados y mayor duración en cualificados.

**Conclusiones:**

Atención primaria asumió el tratamiento en > 90% de los procesos de IT. La derivación a otro nivel asistencial para pruebas o interconsultas puede condicionar una mayor duración de la baja dada la demora habitual del sistema sanitario; el porcentaje de pruebas realizadas en la mutua es bajo. En la duración del proceso se incluyó la observada y la estimada. La duración más larga de la IT corresponde a los trabajadores más cualificados y en trastornos musculoesqueléticos y mentales, pudiendo influir la demora, comorbilidad y factores sociolaborales.

## Abstract (en)

**Objectives:**

To know the characteristics of the sick leave (SL) process, reasons, actual and estimated duration, and related factors.

**Design:**

Descriptive, cross-sectional, retrospective study.

**Setting:**

Health Centers of Burgos's Health Area.

**Participants:**

Active workers, selected by simple random sampling, among those who had a SL process in 2018 and 2019.

**Main measurements:**

Sociodemographic variables; related to SL reasons, estimated and observed duration and job position; clinical variables: habits, comorbidities, polypharmacy and copayment.

**Results:**

Nine hundred thirty-three workers, 52.4% men, age 43.6 ± 11.4 years. The processes with the greatest difference between observed and estimated duration were neoplasms, circulatory system diseases, mental disorders, nervous system and musculoskeletal disorders. Significant difference for previous comorbidities, polypharmacy, no copayment and >55 years. 31.8% requested additional tests, 3.1% were done in mutual insurance companies; and 33.3% consultations. Higher percentage of SL in unskilled jobs and longer duration in skilled jobs.

**Conclusions:**

Primary care assumed the treatment in >90% of the SL processes. Referral to another level of care for tests or consultations may lead to a longer duration of leave given the usual delays in the health system; the percentage of tests carried out at a mutual insurance company is low. The duration of the process included the observed and estimated. The longest duration of SL corresponds to the most qualified workers and to musculoskeletal and mental disorders, and the delay, comorbidity and socio-occupational factors may have an influence.

## Introducción

La incapacidad temporal (IT) es la situación en la que se encuentra un trabajador que, a consecuencia de una enfermedad (común o profesional) o accidente (sea o no de trabajo), está impedido para desempeñar temporalmente su trabajo y requiere asistencia sanitaria^1^.

En los casos de enfermedad común o accidente no laboral el médico responsable de la gestión y del seguimiento en los procesos de hasta 365 días de duración, es el médico de familia de atención primaria. Este profesional es el responsable de realizar una primera valoración clínica, en la que se debe tener en cuenta implicaciones sociales, laborales y económicas, así como realizar el seguimiento clínico continúo del paciente, y finalmente el alta, lo que conlleva una sobrecarga asistencial y burocrática en la consulta diaria^2^. En ocasiones, este proceso complejo se prolonga por las demoras en la realización de las pruebas diagnósticas o en los tratamientos que dependen de otros niveles asistenciales^3^.

En España las IT originan una importante repercusión económica que ha dado lugar a continuas reformas legislativas^4^, viéndose influenciada por los propios cambios socioeconómicos como la crisis económica del 2008^5^ o el incremento del modelo de teletrabajo instaurado por la pandemia COVID-19.

A medida que la economía se fue recuperando de la crisis, a partir del año 2013, la tasa de IT en España ha ido aumentando progresivamente hasta alcanzar un máximo del 4,12% de la población activa en 2019^6^, justo prepandemia.

La incidencia y duración de las IT están ligadas a la empresa y el puesto de trabajo^7^, ^8^, y generan un absentismo, tanto en el sistema público como privado, que da lugar a una alteración en la productividad, competitividad, gestión de personal, etc.^7^.

El Instituto Nacional de la Seguridad Social (INSS) ha editado un manual de tiempos óptimos de IT como una herramienta que permita un mayor respaldo técnico a las actuaciones médicas^9^.

En Burgos según los datos proporcionados por el Plan Estadístico de Castilla y León 2018-2021, el número de bajas tramitadas es superior a la media de la comunidad autónoma, y ocupa el segundo puesto como provincia con mayor incidencia, observándose una tendencia ascendente^10^. Los datos publicados por el Instituto Nacional de la Seguridad Social de IT por contingencias comunes en 2019, ofrece datos de incidencia media mensual por cada 1.000 trabajadores muy dispares entre autonomías, en Castilla y León fue del 17,58, con el máximo valor en Navarra del 43,39 y el mínimo en Extremadura con un 13,55, siendo la media nacional del 24,57. En Europa los sistemas de prestación y gestión de la IT por contingencias comunes son muy diferentes entre países, por lo que son difícilmente comparables, pero es un hecho constatado que España lidera el absentismo laboral.

Los objetivos del estudio son: valorar si los tiempos de duración de la IT estimados por el INSS se ajustan a la duración observada y profundizar en el conocimiento de las comorbilidades, aspectos sociodemográficos y factores laborales que pueden condicionar los motivos, la frecuencia y la duración tanto real como estimada de la IT.

## Material y métodos

### Diseño y población de estudio

Se trata de un estudio transversal y retrospectivo.

El ámbito de estudio es atención primaria, incluyendo los centros de salud rurales y urbanos, del Área de Salud de Burgos.

Los criterios de inclusión fueron los siguientes: trabajador activo con tarjeta sanitaria adscrita al Área de Salud de Burgos y al menos un proceso nuevo de IT por contingencia común entre el 1 de enero de 2018 y 31 de diciembre de 2019 en el periodo estudiado.

Como criterios de exclusión se plantearon: trabajadores afiliados a mutualidades (MUFACE, MUGEJU e ISFAS), trabajadores del régimen general agrario o autónomos, enfermedad profesional y accidentes laborales.

Para una N = 45.576, se calculó un tamaño muestral de 827 personas para una proporción estimada del 27%, nivel de confianza del 95%, precisión del 3%, sumando un posible 15% de pérdidas, teniendo en cuenta que el estudio es retrospectivo. La selección de la muestra se hizo mediante muestreo aleatorio simple sin reemplazo a partir de la relación de personas que cumplía los criterios de inclusión*.*

### Variables analizadas

Los datos se obtuvieron de la historia clínica digital para atención primaria en Castilla y León (MEDORA) que tiene un módulo específico para gestión de procesos de IT por contingencias comunes.

Se contemplaron los siguientes tipos de variables:

**–** Variables sociodemográficas: Edad, sexo, centro de salud. Ámbito: rural o urbano.
– Variables clínicas: Fumador, consumo de alcohol y de drogas. Comorbilidades: diabetes mellitus, hipertensión arterial, EPOC, asma, neoplasias, depresión/ansiedad y otros procesos mentales. Polifarmacia. Aportación farmacéutica. Pruebas diagnósticas, tratamiento e interconsultas en relación con la IT.
– Variables relacionadas con la baja laboral: Fecha y diagnóstico motivo de IT, número de situaciones de IT en el periodo estudiado y en los 5 años previos, duración real de la baja y duración estimada^7^, puesto de trabajo (Clasificación Nacional de Ocupaciones [CNO-11])^11^, actividad profesional (Clasificación Nacional de Actividades Económicas [CNAE])^12^ y relación laboral (sector público o privado).

### Análisis estadístico

Se realizó el análisis descriptivo y comparativo de las variables con el software estadístico SPSS® 28.0. Las variables categóricas se resumieron como frecuencias absolutas y relativas (porcentajes) con intervalos de confianza (IC) del 95% calculado con el método Wilson *score*, con el programa Epi Info v.7.2. Para las cuantitativas se calcularon la media y su IC del 95%, mediana y desviación estándar. Para analizar la relación entre variables cualitativas se ha utilizado la prueba de Chi-cuadrado. Las variables cuantitativas en este estudio no seguían una distribución normal, por lo que se utilizaron como estadísticos de contraste pruebas no paramétricas: la U de Mann-Whitney para comparar 2 grupos y test de Kruskal-Wallis para más de 2 grupos y prueba *post hoc* de Bonferroni en comparación entre pares. La bondad de ajuste a una distribución normal de las variables cuantitativas se evaluó con la prueba de Kolmogorov-Smirnov. Se fijó un nivel de significación del 0,05.

**Figure:**
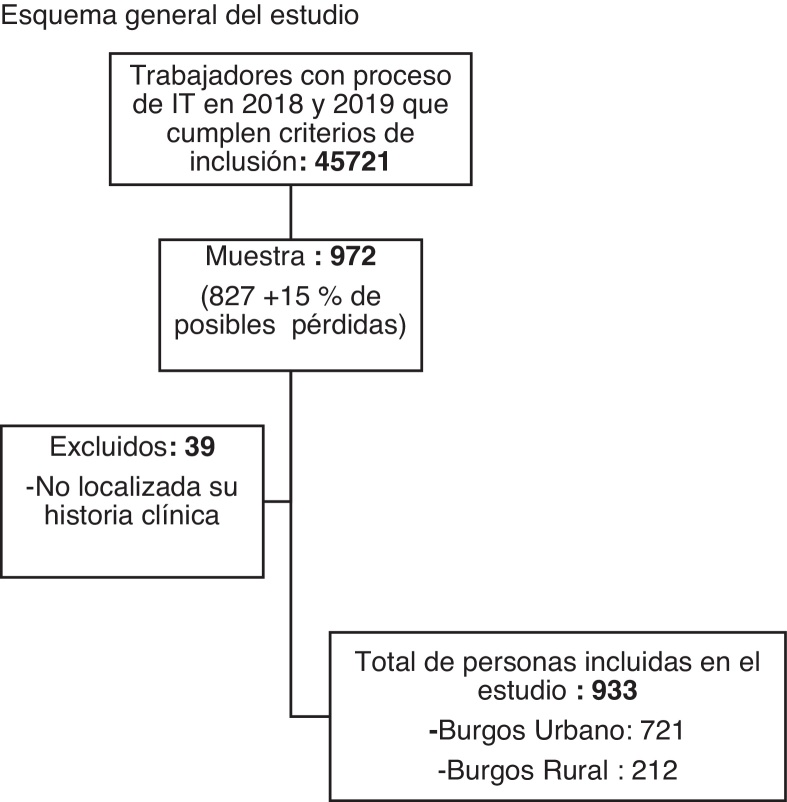
**Esquema del estudio**. Esquema general del estudio.

## Resultados

### Características de la población estudiada

Se incluyeron en el estudio 933 trabajadores con al menos una baja por IT en el bienio. El 52,4% varones y el 47,6% mujeres. La edad media fue de 43,6 ± 11,4 años. No se encontraron diferencias significativas en la frecuencia de episodios de IT por sexo (p = 0,52).

En las variables sobre hábitos de vida, donde existía registro, el 33,1% eran fumadores, el 12,2% consumía alcohol y el 5,6% drogas; el 32,7% tenía sobrepeso y el 29,9% obesidad. Un 27,4% tenía enfermedad psiquiátrica (depresión y/o ansiedad).

### Número y duración de la incapacidad temporal

En el bienio 2018-2019, el 67,5% de los trabajadores tuvieron una sola IT; 2 procesos de IT el 20,0% y más de 2 procesos, el 12,43%.

En los 5 años anteriores el 41,5% no tuvieron otro proceso de IT, un 20,8% habían tenido una IT y un 37,7% ≥ 2 procesos de IT.

El 33,6% fueron episodios de muy corta duración (< 5 días). El 34% de corta duración (5-30 días). El 12,2% duración media (entre 31 y 60 días) y el 20,2% de larga duración (> 60 días).

### Diagnósticos motivo de incapacidad temporal

Los principales procesos motivo de IT se han recogido en la tabla 1. Las enfermedades del aparato musculoesquelético fueron las más frecuentes (24,3%) y dentro de este grupo la enfermedad de la columna vertebral fue el más numeroso (10,1%); en segundo lugar, infecciones respiratorias (18,1%); tercer lugar gastroenteritis (10,9%); cuarto lugar, los trastornos mentales y del comportamiento (7,0%) y dentro de este grupo, fueron los trastornos de ansiedad los más frecuentes como motivo de IT (4,4%).

**Tabla 1:** Procesos más frecuentes y de mayor duración de la baja por incapacidad temporal

		Duración(días) observada			Duración(días) estimada		
Categorías (CIE-10)	N.° (%)	Mediana	Media (DE)	IC 95%	Mediana	Media (DE)	IC 95%
Enfermedades del aparato musculoesquelético	227 (24,3)	22	71,2 (104,3)	57,5-84,8	18	25,8 (31,8)	21,6-29,9
Infecciones agudas respiratorias: resfriado, gripe, faringoamigdalitis, neumonía...	169 (18,1)	4	8,59 (17,3)	6,0-11,2	5	7,1 (9,6)	5,6-8,5
Diarrea; gastroenteritis de posible origen infeccioso	102 (10,9)	2	7,02 (26,8)	1,8-12,3	4	4,5 (6,4)	3,3-5,8
Trastornos mentales y del comportamiento	65 (7,0)	62	84,8 (81,4)	64,6-104,9	29	34,4 (20,8)	29,3-39,6
Enfermedades del ojo y del oído	44 (4,7)	22	34,5 (44,2)	21,1-47,9	13	19,36 (16,9)	14,2-24,5
Enfermedades del aparato circulatorio	32 (3,4)	39,5	91,3 (99,0)	55,7-127,0	24,5	31,7 (21,5)	23,9-39,5
Lesiones traumáticas y otras causas externas	31 (3,3)	18	22,8 (20,9)	15,2-30,5	15	20,48 (16,9)	14,2-26,7
Problemas relacionados con el embarazo	26 (2,8)	34	68,1 (71,4)	39,2-96,9	21,5	34,9 (46,8)	15,9-53,8
Enfermedades del aparato genitourinario	24 (2,6)	9	34,1 (74,8)	2,51-65,7	9,5	15,8 (23,7)	5,8-25,8
Neoplasias	19 (2,0)	104	198 (165,0)	118,5-277,5	113,5	105,4 (90,1)	60,6-150,3
Enfermedades del aparato respiratorio (excepto infección aguda)	16 (1,7)	6,5	21,3 (26,3)	7,2-35,3	9	18,9 (21,7)	7,4-30,5
Enfermedades del sistema nervioso	12 (1,3)	14	80,8 (130,4)	2,8-162,9	21	30,8 (35,6)	8,2-53,5

CIE: Clasificación Internacional de Enfermedades; DE: desviación estándar; IC: intervalo de confianza; IT: incapacidad temporal.

### Pruebas diagnósticas y tratamientos fuera del ámbito de atención primaria

En el 31,8% de los procesos de IT se solicitaron pruebas diagnósticas complementarias en los hospitales de referencia, en el 33,3% se solicitaron interconsultas con especialistas del hospital, y el 9,8% de los procesos de IT requirieron tratamiento hospitalario. En las mutuas se realizaron pruebas diagnósticas en el 3,1% del total de procesos de IT, y tratamiento en la mutua en el 1,9%.

### Duración estimada y observada

**Figura 1:**
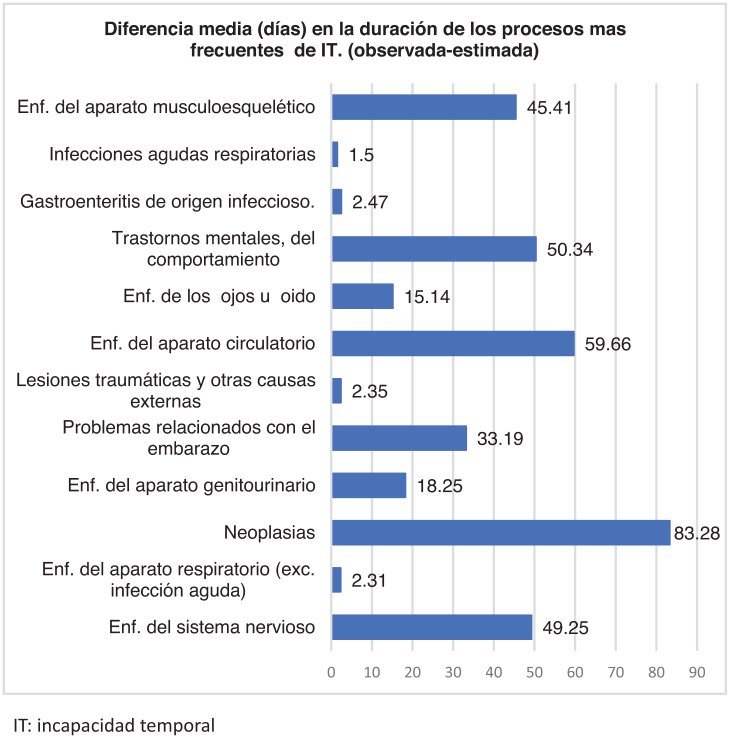
Diferencia media (días) en la duración de los procesos más frecuentes de incapacidad temporal (observada-estimada). IT: incapacidad temporal.

Los procesos con una mayor duración media observada y mayor diferencia entre duración media observada y estimada (tabla 1), se dieron en neoplasias (media de 83,2 días; DE: 164), enfermedades del aparato circulatorio (59,6 días; DE: 90,6), trastornos mentales (50,3 días; DE: 82,4), sistema nervioso (49,2 días; DE: 113,9) y musculoesqueléticas (45,4 días; DE: 99) (fig. 1).

No se apreciaron diferencias significativas por sexo en la duración observada (p = 0,058) ni estimada (p = 0,76). Se obtuvieron diferencias significativas en la edad, tanto para la duración observada (p < 0,001) como en la estimada (p < 0,001), siendo menor la duración en el grupo < 25 años y mayor en el de > 55 años respecto al resto de grupos de edad (tabla 2). La diferencia entre duración observada y estimada solo fue significativa para los > 55 años frente al resto de los grupos (p < 0,02).

**Tabla 2:** Duración de la baja por incapacidad temporal y variables sociodemográficas

	Frecuencia	Duración observada (días)				Duración estimada (días)			
	N.° (%)	Media (DE)	Mediana	IC 95%		Media (DE)	Mediana	IC 95%	
*Sexo*									
Varones	489 (52,4)	41,8 (80,4)	8	34,67-48,95	p = 0,058	22,8 (38,8)	12	19,38-26,29	p = 0,76
Mujeres	444 (47,6)	49,2 (85,2)	14	41,17-57,07		17,1 (25,0)	13	17,08-21,74	

*Edad (años)*									
18-25	52 (5,6)	14,6 (51,2)	3	0,35-28,88	p < 0,001	7,67 (7,5)	4	5,57-9,77	p < 0,001
26-35	197 (21,1)	26,9 (54,0)	6	19,26-34,45		14,9 (26,6)	7	11,20-18,67	
36-45	273 (29,2)	34,5 (67,4)	8	26,49-42,56		16,5 (23,7)	8	13,63-19,28	
46-55	227 (24,3)	48,9 (80,8)	15	38,38-59,52		21,4 (20,4)	15	18,73-24,07	
> 55	184 (19,7)	85,2 (117,3)	35	68,10-102,23		38,6 (54,7)	23	30,65-46,62	

*Mes de inicio*									
Enero	139 (14,9)	29,9 (61,4)	7	19,62-40,24	p = 0,07	14,6 (21,4)	7	11,0-18,18	p = 0,014
Febrero	109 (11,7)	34,5 (71,9)	8	20,80-48,09		22,1 (33,4)	13	15,70-28,39	
Marzo	79 (8,5)	41,1 (64,4)	14	26,69-55,55		19,9 (26,7)	12	13,88-25,84	
Abril	77 (8,3)	54,2 (95,7)	13	32,52-75,93		25,3 (44,7)	14	15,17-35,48	
Mayo	83 (8,9)	54,3 (94,2)	17	33,68-74,82		18,8 (17,1)	15	15,06-22,53	
Junio	70 (7,5)	42,0 (83,0)	8	22,15-61,75		18,7 (21,7)	13	13,45-23,85	
Julio	51 (5,5)	34,8 (54,1)	11	19,54-49,98		21,4 (25,9)	13	14,10-28,68	
Agosto	52 (5,6)	71,4 (115,4)	19,5	39,24-103,48		27,96 (51,7)	18	13,56-42,37	
Septiembre	77 (8,3)	52,1 (85,7)	19	32,64-71,56		26,7 (30,9)	16	19,71-33,75	
Octubre	64 (6,9)	45,3 (78,8)	9	25,55-64,94		27,5 (60,3)	11	12,42-42,58	
Noviembre	65 (7,0)	54,4 (99,5)	12	29,72-79,01		19,48 (24,3)	14	13,46-25,50	
Diciembre	67 (7,2)	53,3 (91,9)	9	30,61-75,44		19,93 (24,5)	13	13,94-25,91	

DE: desviación estándar; IC: intervalo de confianza; IT: incapacidad temporal; p: nivel de significación.

En cuanto a la distribución según mes de inicio de la IT, la mayor incidencia se dio en el primer trimestre del año, pero por procesos de menor duración. La mayor duración observada fue en las IT iniciadas en el mes de agosto, aunque la diferencia no fue significativa (p = 0,07). En los 3 casos, sexo, edad y mes de inicio, la duración observada fue mayor que la estimada.

De la aportación de farmacia, el 91,1% de los trabajadores tuvieron copago, el 6,97% no lo tuvieron y del 0,02% no se dispuso de datos. Fue significativa la diferencia encontrada en ambos grupos, tanto en duración observada (p = 0,004) como estimada (p = 0,003), en ambos casos la duración fue mayor en el grupo sin copago. La diferencia de duración observada/estimada no fue significativa (p = 0,24).

En las variables clínicas (tabla 3), tanto la duración observada como la estimada fue significativamente mayor en trabajadores con antecedentes de hipertensión arterial, neoplasia, depresión o ansiedad y en aquellos con polifarmacia (toma de ≥ 5 fármacos de forma crónica). En los trabajadores con EPOC también fue significativamente mayor la duración observada pero no la estimada.

**Tabla 3:** Duración de la incapacidad transitoria y variables clínicas

		Duración observada de la IT (días)				Duración estimada de la IT (días)			
Total: 933	N.° (%)	Media (DE)	Mediana	IC 95%		Media (DE)	Mediana	IC 95%	
*Fumador*									
No	449 (48,1)	47,2 (83,5)	13	39,4-54,9	p = 0,87	23,2 839,7)	14	19,2-27,3	p = 0,83
Sí	222 (23,8)	52,4 (91,8)	11	40,2-64,6		23,1 (39,7)	13	16,4-29,7	
No consta	262 (28,1)								

*Consumo de alcohol*									
No	511 (54,8)	47,3 (84,9)	12	39,5-55,0	p = 0,31	22,1 (36,6)	13	18,8-25,4	p = 0,27
Sí	71 (7,6)	64,4 (110,5)	18	33,4-95,3		33,3 (60,3)	16	16,3-50,2	
No consta	351 (37,6)								

*Consumo de drogas*									
No	509 (54,6)	49,6 (87,4)	13	41,8-57,4	p = 0,18	23,1 (37,5)	14	19,8-26,5	p = 0,19
Sí	30 (3,2)	37,8 (95,1)	8	0,20-75,4		25,2 (68,8)	7	-2,0-52,4	
No consta	394 (42,2)								

*Diabetes mellitus*									
No	877 (94,0)	48,1 (87,3)	12	40,3-56,0	p = 0,17	22,7 (39,7)	14	19,1-26,3	p = 0,26
Sí	52 (5,6)	61,0 (95,0)	26	27,3-94,8		30,3 (38,8)	15	16,6-44,1	
No consta	4 (0,4)								

*HTA*									
No	802 (86,0)	45,9 (83,6)	11	38,1-53,7	p < 0,001	21,6 (34,4)	13	18,4-24,9	p < 0,001
Sí	129 (13,8)	69,6 (110,2)	21	42,7-96,5		33,6 (63,6)	18	18,1-49,1	
No consta	2 (0,2)								

*EPOC*									
No	916 (98,2)	46,9 (84,9)	12	39,5-54,4	p = 0,016	22,6 (36,9)	14	19,3-25,8	p = 0,14
Sí	16 (1,7)	152,3 (154,7)	85	41,6-262,7		58,4 (116,0)	23	-30,7-147,5	
No consta	1 (0,1)								

*Neoplasia*									
No	885 (94,9)	44,15 (79,9)	11	37,0-51,3	p < 0,001	20,8 (33,5)	13	17,8-23,8	p < 0,001
Sí	47 (5,0)	142,5 (158,5)	66,5	77,1-208,0		72,3 (92,1)	26	33,4-111,3	
No consta	1 (0,1)								

*Depresión/ansiedad*									
No	675 (72,3)	44,3 (82,5)	11	35,8-52,8	p = 0,003	20,2 (32,8)	12	17,7-22,7	p = 0,03
Sí	256 (27,4)	60,9 (99,3)	16	44,5-77,4		23,9 (33,5)	14	19,7-28,0	
No consta	2 (0,2)								

*Polifarmacia, n (%)*									
No	807 (86,5)	44,49 (81,0)	11	37,8-51,1	p < 0,001	21,8 (34,3)	13	18,6-25,0	p = 0,006
Sí	121 (13,0)	75,6 (110,2)	22	53,1-98,2		31,5 (62,2)	14,5	17,0-45,9	
No consta	5 (0,5)								

EPOC: enfermedad pulmonar obstructiva crónica; HTA: hipertensión arterial; IT: incapacidad temporal; p: nivel de significación.

No se obtuvieron diferencias significativas en la duración en el grupo de fumadores o con consumo habitual de alcohol. En consumidores de drogas la duración observada fue menor y la estimada mayor, aunque la diferencia no fue significativa (p = 0,18).

### Relación laboral

En cuanto al puesto de trabajo (tabla 4), el mayor porcentaje de IT se dio en el grupo de operadores de instalaciones y maquinaria, seguido de trabajadores cualificados de industrias manufactureras y trabajadores no cualificados; y una mayor duración en la IT en el grupo de directores y gerentes, seguido de trabajadores de la salud y cuidado de personas y trabajadores de restauración y comercio.

**Tabla 4:** Puesto de trabajo (CNO-11). Frecuencia y duración de la baja por incapacidad temporal

Puesto de trabajo	IT	Duración observada			Duración esperada		
	N.° (%)	Mediana	Media (DE)	IC 95%	Mediana	Media (DE)	IC 95%
Directores y gerentes	14 (1,6)	38,5	74,4 (100,1)	16,5-132,2	21,5	25,6 (17,6)	15,5-35,8
Técnicos y profesionales científicos de la salud y de la enseñanza	59 (6,6)	14	46,6 (72,5)	27,7-65,5	12	16,9 (17,4)	12,4-21,5
Otros técnicos y profesionales científicos e intelectuales	32 (3,6)	8,5	34,0 (69,3)	9,0-59,0	14,5	35,34 (73,4)	8,8-61,8
Técnicos, profesionales de apoyo	49 (5,5)	8	32,6 (63,5)	13,8-50,3	11	16,0 (15,2)	11,6-20,4
Empleados de oficina que no atienden al público	36 (4,0)	4,5	18,0 (26,0)	9,2-26,8	7	17,9 (22,15)	10,4-25,4
Empleados de oficina que atienden al público	58 (6,5)	12	43,7 (75,5)	23,8-63,5	13	19,7 (22,7)	13,7-25,7
Trabajadores de los servicios de restauración y comercio	99 (11,1)	22	66,0 (103,6)	45,3-86,6	14	20,4 (38,4)	12,7-28,0
Trabajadores de los servicios de salud y el cuidado de personas	62 (6,9)	22,5	57,4 (99,1)	32,4-82,3	14,5	17,9 (15,9)	13,8-21,9
Trabajadores de los servicios de protección y seguridad	18 (2,0)	8	58,0 (103,7)	6,4-109,6	12	23,7 (32,5)	7,6-39,9
Trabajadores cualificados en el sector agrícola, ganadero, forestal y pesquero	13 (1,5)	24	80,8 (128,5)	3,2-158,5	29	49,7 (53,6)	17,2-82
Artesanos y trabajadores cualificados de las industrias manufactureras y la construcción	105 (11,8)	8	52,0 (96,0)	33,5-70,6	11	23,1 (32,3)	16,8-29,3
Operadores de instalaciones y maquinaria, montadores. Conductores y operadores de maquinaria móvil	158 (17,7)	6	37,7 (76,6)	25,6-49,7	8	22,4 (43,2)	15,6-29,1
Trabajadores no cualificados en servicios, empleados domésticos, vendedor callejero	100 (11,2)	11,5	48,3 (84,4)	31,6-65,0	14,5	22,3 (26,4)	17,0-27,6
Peones de la agricultura, pesca, construcción, industrias manufactureras y transportes	90 (10,1)	8	29,36 (58,5)	17,1-41,6	12	16,9 (16,6)	13,5-20,4

CNO: clasificación nacional de ocupaciones; DE: desviación estándar; IC: intervalo de confianza; IT: incapacidad temporal.

El 81,9% trabajaban en el sector privado y el 14% en el público, en el 4,1% no constaba. El porcentaje de trabajadores con IT por actividad empresarial fue superior al esperado, según el porcentaje de trabajadores de cada sector (tabla 5) del Área de Salud de Burgos, en los grupos de: actividades de construcción, el 5,27% de trabajadores en ese grupo y el 9,46% del total de IT; actividades financieras y de seguro, con el 1,64% de trabajadores de ese grupo y el 3,04% del total de IT; en otros servicios, con el 1,82% de trabajadores del grupo y el 3,15% de IT y en el grupo de actividades del hogar con el 0,35% de los trabajadores y el 1,58% del total de IT.

**Tabla 5:** Número de trabajadores por empresas y porcentaje de incapacidad temporal

		Trabajadores		Bajas laborales por IT		
Grupo empresa (CNAE)		N.°	%	N.°	%	IC 95%^a^
A	Agricultura, ganadería y pesca	809	0,70	21	2,36	1,55-3,59
B	Industrias extractivas (extracción de minerales, grava, piedra, sal…)	416	0,36	3	0,34	0,11-0,99
C	Industria manufacturera: alimentación, bebidas, vehículos de motor…	29.055	25,27	228	25,68	22,91-28,65
D	Suministro de energía eléctrica, gas vapor y aire acondicionado	461	0,40	18	2,03	1,29-3,18
E	Suministro de agua, actividades de saneamiento, gestión de residuos	1.068	0,93	6	0,68	0,31-1,47
F	Actividades de construcción	6.055	5,27	84	9,46	7,71-11,56
G	Comercio al por mayor y al por menor. Venta y reparación de vehículos…	15.034	13,07	82	9,23	7,4-11,20
H	Actividades de transporte y almacenamiento. Taxis, autobuses…	5.330	4,64	45	5,07	3,81-6,71
I	Actividades de hostelería	8.225	7,15	56	6,31	4,89-8,1
J	Actividades de información y comunicaciones	972	0,85	8	0,90	0,38-1,62
K	Actividades financieras y de seguros. Banca, seguros, etc.	1.882	1,64	27	3,04	2,1-4,39
L	Actividades inmobiliarias. Compraventa, inmobiliarias. Agentes de la propiedad	376	0,33	1	0,11	0,02-0,64
M	Actividades profesionales, científicas y técnicas	3.757	3,27	32	3,60	2,56-5,04
N	Actividades administrativas y servicios auxiliares. Administrativos, jardinería, limpieza, agencias de viaje, centros de llamadas telefónicas, envasado y empaquetado	8.491	7,38	45	5,07	3,81-6,71
O	Administración pública y defensa. orden público, protección civil	5.816	5,06	48	5,41	4,1-7,09
P	Actividades de educación	8.322	7,24	29	3,27	2,28-4,65
Q	Actividades sanitarias y de servicios sociales, establecimientos de personas mayores	14.820	12,89	106	11,94	9,97-14,24
R	Actividades artísticas, recreativas y de entretenimiento	1.608	1,40	7	0,79	0,38-1,62
S	Otros servicios (organizaciones empresariales, sindicales, asociativas, reparación de artículos, peluquería, pompas fúnebres, etc.)	2.091	1,82	28	3,15	2,19-4,52
T	Actividades del hogar. Empleador de personal doméstico	405	0,35	14	1,58	0,94-2,63

CNAE: Clasificación Nacional de Actividades Económicas; IT: incapacidad temporal.

a Wilson *score*.

## Discusión

En cuanto a las variables clínicas, destaca la elevada prevalencia de obesidad y hábito tabáquico, respecto a los datos nacionales publicados en la Encuesta Europea de Salud en España de 2020, así como de enfermedad psiquiátrica, si bien estos datos son poblacionales y los del estudio, de trabajadores en activo.

### Duración y frecuencia de la incapacidad temporal

Nuestros hallazgos coinciden con otros estudios, en los principales diagnósticos de motivos de IT y de mayor duración^10^, ^13^, ^14^; y en que a mayor edad mayor frecuencia^18^ y duración de la IT^13^, ^15^, ^16^, ^17^ observada. En nuestro caso esta relación también se da con la duración estimada, lo que se justificaría por una mayor comorbilidad y cronicidad de los procesos motivo de IT a mayor edad, parámetros tenidos en cuenta por el INSS.

Sin embargo, no encontramos en el presente estudio diferencias significativas por sexo en la frecuencia ni duración, aunque otros trabajos^5^, ^13^, ^15^, ^16^, ^17^ concluyeron que es superior en las mujeres.

Un tercio de los casos precisaron derivación a otro nivel asistencial para pruebas o interconsulta, lo que puede condicionar en gran medida una mayor duración de la baja dada la demora habitual en el sistema sanitario^14^, ^19^. El porcentaje de pruebas realizadas en la mutua es bajo, sería conveniente una mayor colaboración con las mutuas para agilizar estos procedimientos. Cabe destacar que atención primaria asumió el tratamiento en más del 90% de los procesos de IT.

Así mismo, la duración excesiva se relaciona no solo con la variabilidad clínica y la comorbilidad sino también con factores de difícil abordaje psicológicos^14^, socioeconómicos y la cultura extendida de uso de la IT para otros fines^19^, ^20^ (paro, ceses, problemas familiares, conflictos laborables^8^, ^15^, etc.). Es conocido que el 20% de las IT son responsables del 80% del gasto^19^.

### Duración de la incapacidad temporal observada y estimada

Hallamos una mayor duración tanto observada como estimada, de forma significativa, en los pacientes con hipertensión arterial, neoplasias, depresión o ansiedad; del mismo modo sucedió con el no copago en farmacia, medidor indirecto de bajo nivel socioeconómico. En la EPOC no fue estadísticamente significativa la diferencia, tampoco en la duración asociada a estilos de vida, aunque se debe ser cauteloso con su interpretación ya que en ambos casos tuvimos muchos datos perdidos, de hecho, en otros estudios si se encontró asociación^21^, ^22^.

En este estudio, la duración más larga de la IT corresponde a los trabajadores con mayor cualificación, directivos y trabajadores de la salud, que quizás traduzca un mayor estrés laboral^8^, ^23^, ya que se ha asociado con un mayor número de IT por trastornos mentales^24^.

En los procesos de corta duración encontramos menor la duración observada que la estimada. En la mayoría de los diagnósticos hay un exceso de días entre la duración observada y estimada en casos de media y larga duración; esto puede explicarse por la variabilidad clínica en casos como las neoplasias y enfermedades del aparato circulatorio, pero menos en los trastornos del aparato musculoesquelético y trastornos mentales (depresión y/o ansiedad), donde probablemente pueda tener más influencia la demora del sistema sanitario^14^, factores sociolaborales^8^, ^25^, un posible rédito^20^ y la duración del proceso^26^, ^27^. En los trastornos mentales la existencia de comorbilidad^28^ y sobre todo el antecedente de trastorno mental^29^ son factores que se asocian con una mayor duración de la IT, así como cuando el convenio colectivo mejora la cuantía a percibir y el pago es mayor^30^. Esto hace que la negociación colectiva y una adecuada prevención de riesgos laborales pudieran ser herramientas realmente eficaces en la lucha contra el absentismo^7^.

### Limitaciones

El diseño del estudio imposibilita establecer relaciones de causalidad. Al ser un estudio retrospectivo y basado en los datos de las historias clínicas hay perdida de información, más evidente en variables relacionadas con estilos de vida como consumo de tabaco, alcohol y drogas, que se consideraron valores perdidos para el análisis estadístico. La dispersión de los datos de alguna variable, en la que además se dispone de pocos casos, condiciona poder extraer conclusiones con mayor evidencia, pese a que se incrementó el tamaño muestral para minimizar su impacto.

Para minimizar los posibles sesgos de selección, se incrementó el tamaño muestral y se tuvo en cuenta que el fichero de la muestra incluyera el total de trabajadores con al menos un proceso de IT en el periodo estudiado y que cumplieran los criterios de selección.

Aunque los criterios de exclusión podrían limitar la generalización de los hallazgos obtenidos, sin embargo, permiten obtener una imagen más real de la IT en las personas asalariadas, evitando posibles sesgos, en aquellos colectivos, como los autónomos, con situación sociolaboral especial que condiciona las bajas laborales o en el caso de los accidentes laborales o enfermedades profesionales y las mutualidades, cuya gestión de las bajas laborales no participan, en la mayoría de los casos, los médicos de atención primaria del Sistema Público de Salud.

Es necesario hacer estudios prospectivos que permitan poder precisar las causas que se asocian a la mayor duración de la IT y revisar las tablas de tiempos estimados del INSS. En ellos, mediante entrevista personal, se podrían incluir variables sobre la percepción subjetiva de la situación de IT por el paciente, estrés y ambiente laborales, que enriquecieran cualitativamente los datos objetivos.

El disponer de datos del periodo 2018 y 2019, prepandemia, nos permitirá evaluar en sucesivos estudios, la influencia de la crisis sanitaria.

**Lo conocido sobre el tema**

• La gestión de la IT por contingencias comunes recae en el médico de familia, siendo un proceso complejo, no solo clínico, y con importante carga asistencial y burocrática.
• En la última década se ha ido incrementando anualmente el número de procesos de IT, con importante repercusión a nivel laboral y económico.
• La IT con una duración excesiva es debida a la propia enfermedad, la demora atribuible al sistema sanitario, y otras causas de difícil abordaje como la comorbilidad, psicológicas, socioeconómicas y la cultura poblacional extendida de uso de la IT, para otros fines (paro, ceses, conflictos laborables, familiares, etc.).

**¿Qué aporta este estudio?**

• Determina de los procesos de IT la diferencia entre la duración observada y la estimada según el INSS, lo que puede ayudar en su gestión.
• Considera la comorbilidad y aspectos sociolaborales relacionados con el proceso de la IT, y que influyen en su duración.
• Describe las pruebas diagnósticas, tratamiento e interconsultas solicitadas en relación con la IT, lo que puede ser una oportunidad de mejora, en colaboración con las mutuas, para minorar las demoras del sistema sanitario.

## Financiación

No se ha recibido financiación específica para la realización de este estudio. Se ha recibido financiación de la Confederación de Asociaciones Empresariales de Burgos (FAE), a través de la Fundación Científica Colegio de Médicos de Burgos, para su publicación en una revista *Open Access.*

## Consideraciones éticas

El trabajo se ha llevado a cabo de conformidad con el código de ética (Declaración de Helsinki actualizada) y siguiendo las recomendaciones de la Guía de Buenas Prácticas en investigación en atención primaria. El protocolo fue aprobado por el Comité de Ética de Investigación con medicamentos (CEIm) del Área de Salud de Burgos y Soria (Ref. CEIm 2803) con fecha 26-07-2022 y autorizada su realización por la Gerencia de Atención Primaria de Burgos con fecha 10-10-2022. La selección de la muestra se hizo a partir del fichero facilitado por la Gerencia Regional de Salud de Castilla y León con personas que cumplían los criterios de inclusión, identificadas únicamente con el código de identificación personal de la tarjeta sanitaria, cuyo acceso estaba únicamente disponible para los investigadores del proyecto. El acceso a la historia clínica informatizada de los pacientes se realizó mediante la selección de la opción «fines de investigación», sin intervención, tal como figuraba en el protocolo aprobado. Se han utilizado los datos anonimizados y exclusivamente para los objetivos del estudio, estando protegidos por la Ley Orgánica 3/2018, de 5 de diciembre, de Protección de Datos Personales y garantía de los derechos digitales y por el Reglamento (UE) 2016/679 del Parlamento Europeo y del Consejo, de 27 de abril de 2016, relativo a la protección de las personas físicas en lo que respecta al tratamiento de datos personales.

## Conflicto de intereses

Los autores declaran no tener ningún conflicto de intereses.
